# *Ginkgo biloba* extract reduces high-glucose-induced endothelial adhesion by inhibiting the redox-dependent interleukin-6 pathways

**DOI:** 10.1186/1475-2840-11-49

**Published:** 2012-05-03

**Authors:** Jia-Shiong Chen, Yung-Hsiang Chen, Po-Hsun Huang, Hsiao-Ya Tsai, Yuh-Lien Chen, Shing-Jong Lin, Jaw-Wen Chen

**Affiliations:** 1 Institute of Pharmacology, National Yang-Ming University, Taipei, Taiwan, Republic of China; 2 Graduate Institute of Integrated Medicine, and Graduate Institute of Clinical Medical Science, China Medical University and Hospital, Taichung, Taiwan, Republic of China; 3 Institute of Clinical Medicine, and Cardiovascular Research Center, National Yang-Ming University, Taipei, Taiwan, Republic of China; 4 Division of Cardiology, Taipei Veterans General Hospital, Taipei, Taiwan, Republic of China; 5 Institute of Anatomy and Cell Biology, School of Medicine, National Taiwan University, Taipei, Taiwan, Republic of China; 6 Department of Medical Education and Research, Taipei Veterans General Hospital, No. 201, Sec. 2, Shih-Pai Road, Taipei, 11217, Taiwan, Republic of China

**Keywords:** Antioxidant, Endothelial cells, *Ginkgo biloba* extract, Glucose, Intercellular adhesion molecule −1, Interleukin −6

## Abstract

**Background:**

Chronic elevation of glucose level activates vascular inflammation and increases endothelial adhesiveness to monocytes, an early sign of atherogenesis. This study aimed to elucidate the detailed mechanisms of high-glucose-induced endothelial inflammation, and to investigate the potential effects of *Ginkgo biloba* extract (GBE), an antioxidant herbal medicine, on such inflammation.

**Materials and methods:**

Human aortic endothelial cells were cultured in high glucose or mannitol as osmotic control for 4 days. The expression of cytokines and adhesion molecules and the adhesiveness of endothelial cells to monocytes were examined. The effects of pretreatment of GBE or *N*-acetylcysteine, an antioxidant, were also investigated.

**Results:**

Either high glucose or mannitol significantly increased reactive oxygen species (ROS) production, interleukin-6 secretion, intercellular adhesion molecule-1 (ICAM-1) expression, as well as endothelial adhesiveness to monocytes. The high-glucose-induced endothelial adhesiveness was significantly reduced either by an anti-ICAM-1 antibody or by an interleukin-6 neutralizing antibody. Interleukin-6 (5 ng/ml) significantly increased endothelial ICAM-1 expression. Piceatannol, a signal transducer and activator of transcription (STAT) 1/3 inhibitor, but not fludarabine, a STAT1 inhibitor, suppressed high-glucose-induced ICAM-1 expression. Pretreatment with GBE or *N*-acetylcysteine inhibited high-glucose-induced ROS, interleukin-6 production, STAT1/3 activation, ICAM-1 expression, and endothelial adhesiveness to monocytes.

**Conclusions:**

Long-term presence of high glucose induced STAT3 mediated ICAM-1 dependent endothelial adhesiveness to monocytes via the osmotic-related redox-dependent interleukin-6 pathways. GBE reduced high-glucose-induced endothelial inflammation mainly by inhibiting interleukin-6 activation. Future study is indicated to validate the antioxidant/anti-inflammatory strategy targeting on interleukin-6 for endothelial protection in *in vivo* and clinical hyperglycemia.

## Introduction

In the early stages of atherogenesis, the adhesiveness of vascular endothelium to monocytes is increased, which may be mediated by endothelial expression of adhesion molecules such as intercellular adhesion molecule 1 (ICAM-1), vascular cell adhesion molecule 1 (VCAM-1), and others as a result of endothelial dysfunction [1]. Clinical evidence suggests that hyperglycemia is an independent risk factor of diabetes-associated atherosclerosis [2]. In vitro studies also suggest that high concentration of glucose (high glucose) could induce ICAM-1 expression [3] and increase the production of chemokines such as interleukin-6 (IL-6) through a tyrosine kinase mechanism involving the Janus kinase/signal transducer and activator of transcription (JAK/STAT) pathways [4]. However, the detailed mechanisms by which high glucose could induce endothelial adhesiveness have not been fully clarified.

It was suggested that leukocyte-endothelial interaction may be augmented by high glucose or hyperglycemia in a NF-kB-dependent fashion [5]. It was also reported that high glucose might induce ICAM-1 accumulation by a non-specific osmotic effect [6]. On the other hand, VCAM-1 expression was shown to increase in endothelial cells by serum from type 1 diabetic patients but not by high-glucose stimulation [7]. Previous studies further indicated that the short-term presence of inflammatory cytokines rather than high glucose could induce endothelial expression of VCAM-1 and ICAM-1 [8]. Accordingly, the mechanisms of high-glucose-induced endothelial expression of adhesion molecules could be complex. It was not known whether and how inflammatory cytokines could alter endothelial expression of adhesion molecules in the presence of high glucose.

*Ginkgo biloba* extract (GBE), an herbal medicine with antioxidant mechanisms, has been used as a therapeutic agent for some cardiovascular and neurological disorders [9]. Although the exact mechanism has not been completely clarified, cumulative *in vitro* and *in vivo* evidence suggests the protective effects of GBE in ischemia/reperfusion injury [10] by reducing oxidative stress [11]. Our previous studies also showed that GBE may inhibit neointimal hyperplasia [12] and cytokine-stimulated endothelial adhesiveness to monocytes [13], at least in part by inducing heme oxygenase-1 to reduce intracellular oxidative stress [14]. However, it was not known whether and how GBE could reduce high-glucose-induced endothelial adhesiveness.

Clinical evidence suggests that endothelial adhesion molecule expression is enhanced in the aorta and the internal mammary artery of diabetic patients [15]. Additionally, hyperglycemia-mediated adhesion of neutrophils to rat endothelial cells could be attenuated by superoxide dismutase *in vivo*[16]. These findings support the hypothesis that intracellular oxidative stress might be critical to high-glucose-mediated, diabetes-associated atherosclerosis. The current study was conducted 1) to clarify whether and how redox-related mechanisms could contribute to high-glucose-induced endothelial adhesiveness to monocytes, and 2) to investigate whether GBE as an herbal antioxidant could reduce *in vitro* endothelial adhesiveness in the presence of high glucose. Our findings may help to further clarify the complex mechanisms by which high glucose might induce endothelial inflammation and to provide the rationale to the potential role of anti-inflammatory strategy for vascular protection in clinical hyperglycemia.

## Materials and methods

This in vitro study was approved by the research committee of Taipei Veterans General Hospital, Taipei, Taiwan, Republic of China.

### Reagents

Endothelial cell growth medium (M200) was obtained from Cascade Biologics (Cascade Biologics, Portland, OR). The GBE (Cerenin) was purchased from Dr. Willmar Schwabe (Dr. Willmar Schwabe, Inc.). 2′,7′-bis(2-carboxyethyl)-5(6)- carboxyfluorescein acetoxymethyl ester (BCECF-AM) and 2′,7′- dichlorofluorescein diacetate (DCFH-DA) were obtained from Molecular Probes (Invitrogen/Molecular Probes). Antibodies against human ICAM-1 and β-actin were obtained from R&D Systems (R&D Systems) and Chemicon (*Chemicon*, Temecula), respectively. Unless otherwise specified, all chemicals and reagents were from Sigma (Sigma).

### Cell cultures and cell viability assay

HAECs (Cascade Biologics) and THP-1 from American Type Culture Collection (Rockville) were cultured as described previously [14]. After incubation with glucose, mannitol, or GBE, cell viability was always found to be greater than 95% by using the trypan blue exclusion method or a 3-(4,5-dimethylthiazol-2- yl)-2,5-diphenyl tetrazolium bromide (MTT) assay (Sigma).

### Monocyte-endothelial cell adhesion assay

The adherence of THP-1, a human monocytic cell line, to glucose- and mannitol-activated HAECs was examined under static conditions to explore the endothelial cell–leukocyte interaction. Adhesion assays were performed as described previously [14]. HAECs were grown to sub-confluence in 24-well plates and treated with glucose or mannitol for 3 days, followed by treatment with GBE or NAC for 1 day.

### Western blot analysis

Western blot analyses were performed using the methods as described previously [14].

### Enzyme-linked immunosorbent assay (ELISA)

HAECs were continuously incubated in a high-glucose medium with or without antioxidants for 4 days. The levels of secreted IL-6 in the medium were determined by ELISA with the human IL-6 kit (BioSource). The procedures were carried out according to the instructions of the manufacturer.

### Electrophoretic mobility shift assay (EMSA)

EMSA was performed as previously described [13]. The synthetic, double-stranded oligonucleotides used in the gel shift assay as the STAT1 and STAT3 probes were 5′-GAT CTT CAG TTT CAT ATT ACT CTA AAT CCA GGA TC-3′ and 5′-GAT CCC TTC GGG AAT TCC TAG ATC-3′, respectively. For supershift analyses, anti-STAT1 and anti-STAT3 monoclonal antibodies (Santa Cruz) were added to the reaction mixture 60 min before the addition of labeled oligonucleotides at 4°C.

### Measurement of ROS production

ROS production in HAECs was determined by a fluorometric assay using DCFH-DA as a probe to detect the presence of H_2_O_2_. The fluorescence intensity (RFU) was measured at an excitation wavelength of 485 nm and an emission wavelength of 530 nm by using a fluorescent microplate reader (VICTPR2 Multilabel Readers, USA).

### Statistical analysis

All data were expressed as the mean ± SEM. Intergroup comparisons were performed by using a Student’s *t* test or one-way analysis of variance (ANOVA). A *p value* < 0.05 was considered statistically significant.

## Results

### High glucose time-dependently increases ICAM-1 and IL-6 expression in HEACs

**Figure 1:**
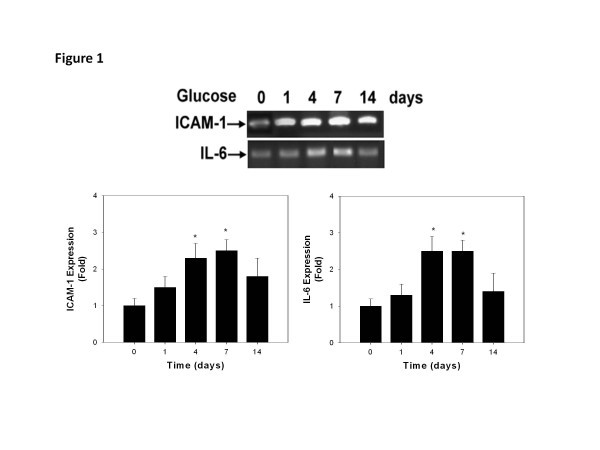
**Time-dependent effects of high glucose (D-glucose, 25 mmol/l) on ICAM-1 & IL-6 expression in human aortic endothelial cells.** Western blotting analyses (upper panel) showed that both ICAM-1 (left lower panel) and IL-6 expression (right lower panel) were significantly increased after high glucose treatment for 4 days through 7 days and reduced after 14 days. N = 4 in each set of experiment. * *p* < 0.05 compared to day 0.

It has not been clarified which adhesion molecules such as ICAM-1, VCAM-1 or both and which cytokines might be involved in glucose-induced monocyte adhesion to endothelial cells [17]. Thus, we first examined the chronic time-dependent effects of high glucose (D-glucose, 25 mmol/l) on the expression of some adhesion molecules including ICAM-1 and VCAM-1 and inflammatory cytokines including IL-1, IL-6, and TNF-alpha in HAECs. Western blotting analyses showed that both ICAM-1 and IL-6 expression were significantly increased after high glucose treatment for 4 days through 7 days and reduced after 14 days (Figure 1). There were no significant changes of endothelial VCAM-1, IL-1, and TNF-alpha expression by high glucose treatment for up to 14 days in this in vitro experiment (Data not shown).

### Long-term effects of high glucose and mannitol on endothelial cell viability, ICAM-1 expression, and adhesiveness to monocytes

**Figure 2:**
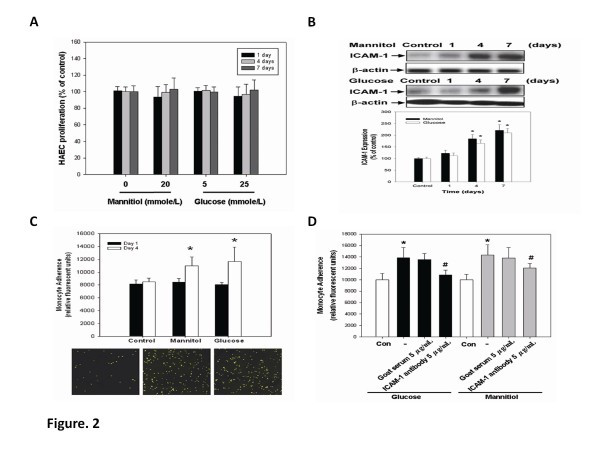
**Cell viability reduction, ICAM-1 expression, and monocyte–endothelial cell adhesion mediated by high glucose in HAECs. (A)** HAECs were treated with d-glucose (25 mM) or osmotic effect control mannitol (20 mM) for 4 days. **(B)** HAECs were incubated in glucose for 0, 1, 4, and 7 days. Cell lysates of ICAM-1 were determined by western blotting. **(C)** THP-1 was labeled with BCECF-AM and adhered to HAECs in response to normal-glucose, high-glucose, and mannitol treatment for 4 days. **(D)** HAECs in response to high-glucose and mannitol treatment for 4 days followed by neutralization with ICAM-1 antibody for 30 min (5 μg/ml). N = 8 in each set of experiment. *p < 0.05 compared to control group; #*p* < 0.05 compared to high-glucose or mannitol groups.

An MTT assay was conducted to investigate the cytotoxicity effect of high glucose and mannitol, as the osmotic control, in cultured HAECs. Exposure to d-glucose (25 mmol/l) or mannitol (25 mmol/l) for 1, 4, and 7 days resulted in no cytotoxicity in HAECs (Figure 2A). Western blotting analysis showed that treatment with high glucose or mannitol time-dependently increased ICAM-1 expression in HAECs (Figure 2B). However, the effect of high glucose stimulation was reversible since the high-glucose-induced expression of ICAM-1 was being reduced 1 to 4 days after the high-glucose medium was replaced by normal medium (Additional file 1: Figure S3). Further, as shown in Figure 2C, long-tem exposure of HAECs to high glucose for 4 days significantly increased endothelial adhesiveness to THP-1 cells, which was similar to the effects of mannitol. The results suggest a common osmotic effect that might contribute to the induction of endothelial ICAM-1 as well as endothelial adhesiveness by high glucose or mannitol. To investigate the role of ICAM-1 expression in high-glucose-induced endothelial adhesiveness to monocytes, glucose-stimulated HAECs were incubated with an anti-ICAM-1 antibody at 5 μg/ml for 30 minutes prior to the adhesion assay. As shown in Figure 2D, the adhesion of THP-1 cells to high-glucose-stimulated HAECs was significantly suppressed by anti-ICAM-1 antibody (21.4% inhibition, *p* < 0.05), but not by a nonspecific goat serum IgG antibody. Taken the above together, it suggested that long-term exposure to high glucose, probably via the osmotic effects, may enhance endothelial adhesiveness to monocytes by increasing endothelial ICAM-1 expression.

### IL-6 contributes to high glucose- or mannitol-induced endothelial adhesiveness to monocytes

**Figure 3:**
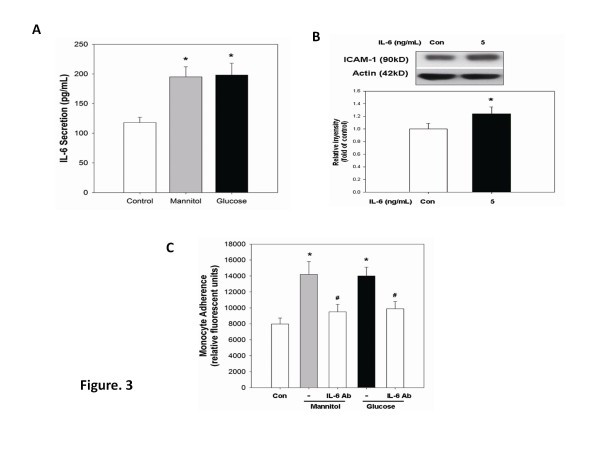
**IL-6 production contributes to glucose-induced monocyte adhesiveness to HAECs. (A)** HAECs were responsive to high glucose and mannitol for 4 days. The conditioned medium was isolated, and IL-6 expression was determined by ELISA. **(B**) HAECs were incubated with 5 ng/ml of IL-6 cytokine for 24 h. The presence of ICAM-1 in cell lysates was determined by western blotting. **(C)** HAECs were responsive to high glucose and mannitol for 3 days, followed by treatment with IL-6 antibody for 1 day (5 μg/ml). N = 6 in each set of experiment. *p < 0.05 compared to control group; #*p* < 0.05 compared to high-glucose or mannitol groups.

In the present study, long-term incubation with high glucose increased IL-6 production, which may increase monocyte-endothelial interaction [18]. In this study, incubation of HAECs with high glucose or mannitol for 4 days similarly and significantly increased IL-6 protein secretion in the medium (Figure 3A). Furthermore, exposure to IL-6 (5 ng/ml) significantly increased ICAM-1 protein accumulation by 24% (*p* < 0.05) in cultured HAECs (Figure 3B), which was compatible to the previous suggestion that IL-6 may mediate monocyte adhesion to activated endothelial cells [19]. Furthermore, to inhibit the effects of endothelial-derived IL-6, the IL- 6 neutralizing antibody was added to high-glucose treated HAECs, which significantly reduced endothelial adhesiveness to monocytes by 57.7% (*p* < 0.05) (Figure 3C), suggesting the crucial role of endothelial-derived IL-6 in either high-glucose or mannitol-induced endothelial adhesiveness to monocytes.

### GBE inhibits ROS generation, IL-6 and ICAM-1 expression, and the adhesiveness to monocytes in high-glucose- or mannitol-stimulated HAECs

**Figure 4:**
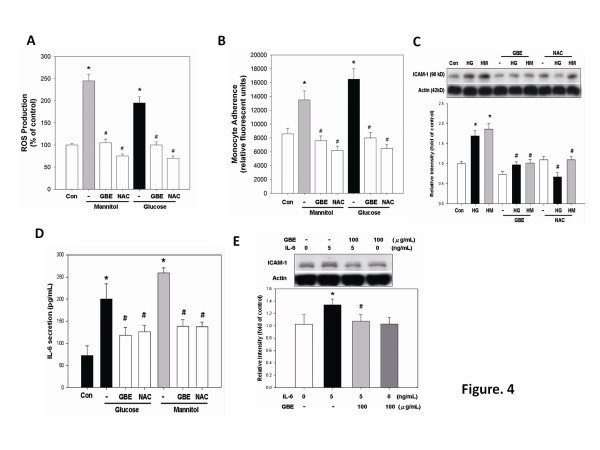
**GBE inhibits high-glucose-induced ROS generation, IL-6 and ICAM-1 expression, and monocyte adhesiveness to HAECs. (A)** HAECs were incubated in high glucose or mannitol for 3 days, followed by incubation with GBE (100 μg/ml) or NAC (1 mmol/l), an antioxidant agent, for 1 day. **(B)** THP-1 labeled with BCECF-AM adhered to HAECs in response to high-glucose or mannitol treatment for 3 days, followed by addition of GBE or NAC for 1 day. **(C)** HAECs were incubated in high glucose or mannitol for 3 days, followed by incubation in GBE and NAC for 1 day. **(D)** HAECs were incubated in glucose or mannitol for 3 days, followed by incubation with GBE or NAC for 1 day. Human IL-6 secreted into the medium was measured using ELISA. **(E)** HAECs were incubated with GBE for 30 min, followed by treatment with IL-6 (5 ng/ml) cytokine for 24 h. The presence of ICAM-1 was determined by western blotting. N = 6 in each set of experiment. *p < 0.05 compared to control group; #*p* < 0.05 compared to high-glucose or mannitol groups.

It has been suggested that hyperglycemia may induce endothelial ROS generation [20]. *N*-acetylcysteine (NAC), an antioxidant, was shown to abolish high-glucose-induced oxidative stress and adhesion molecule expression in endothelial cells [21]. In the present study, intracellular ROS generation was significantly increased by high glucose, which could be inhibited by pretreatment with GBE (100 μg/ml) or NAC (1 mmol/l) (Figure 4A). On the other hand, pretreatment with GBE (100 μg/ml) or NAC (1 mmol/l) for 1 day significantly inhibited the adhesion of THP-1 cells to high-glucose- or mannitol-activated HAECs (Figure 4B). Western blot and ELISA study also showed that pretreatment with GBE (100 μg/ml) or NAC (1 mmol/l) significantly and similarly suppressed glucose-induced endothelial ICAM-1 expression (Figure 4C) and IL-6 secretion in the conditioned medium (Figure 4D) respectively. Finally, GBE directly inhibited IL-6-induced ICAM-1 expression in HAECs (Figure 4E). These findings indicate that GBE, probably by its antioxidant capacity, may inhibit high-glucose-activated IL-6 secretion and consequent ICAM-1-mediated endothelial adhesiveness to monocytes.

### STAT3 is involved in high-glucose- and mannitol-induced ICAM-1 accumulation in HAECs

Several transcription pathways may be involved in the activation of ICAM-1 in endothelial cells. Our preliminary data showed that both AP-1 and NF-κB could be activated with increased nuclear expression by high glucose or by mannitol in HAECs. However, while significantly reduced ICAM-1 expression, treatment with GBE did not significantly modify the activation of either AP-1 or NF-κB in the presence of high glucose or mannitol ( Additional file 1: Figure S1). It seems that other transcription pathways may be more relevant to ICAM-1 induction in current study model.

**Figure 5:**
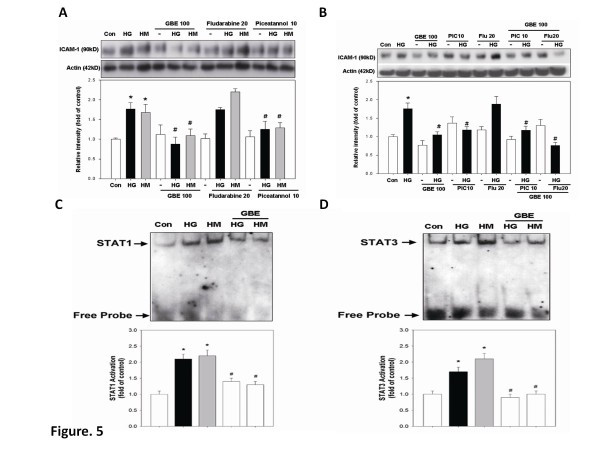
**GBE inhibits high-glucose-induced activation of STAT1 and STAT3 as well as ICAM-1 accumulation in HAECs. (A)** HAECs were incubated in high glucose for 3 days, followed by incubation with GBE, fludarabine (STAT1 inhibitor;20 μmol/l), or piceatannol (10 μmol/l) for 1 day. **(B)** HAECs were incubated in high glucose for 3 days, followed by incubation with GBE, fludarabine, piceatannol, fludarabine plus GBE, or piceatannol plus GBE for 1 day. **(C and D)** EMSAs for STAT1 and STAT3 were performed using nuclear extracts from human aortic endothelial cells that were cultured in high glucose or mannitol for 3 days, followed by treatment with GBE. Quantification of STAT1 and STAT3 activation in HAECs treated with high glucose combined with GBE is also shown. N = 6 in each set of experiment. *p < 0.05 compared to control group; # *p* < 0.05 compared to the high-glucose or mannitol groups.

Another principle factor of this regulatory mechanism is the transcription factor STAT3, which forms either homodimers or heterodimers with STAT1 and translocates into the nucleus [4]. In this study, treatment with piceatannol, a STAT1/3 inhibitor, but not fludarabine, a STAT1 inhibitor, could significantly suppress glucose-induced protein expression of ICAM-1 (Figure 5A). On the other hand, the combination of fludarabine but not piceatannol with GBE significantly suppressed glucose-induced ICAM-1 expression (Figure 5B). The above findings suggest that STAT3 might play the crucial role in high-glucose-induced ICAM-1 expression in HAECs.

### GBE inhibits high-glucose-induced activation of STAT1 and STAT3

It was suggested that redox-related transcriptional regulation may be involved in the activation of STAT1/3 for the induction of ICAM-1 genes [22]. The results of EMSAs indicated that treatment with high glucose or mannitol resulted in the band shifts of both STAT1 and STAT3. These band shifts were specific for STAT1 and STAT3 binding because they were undetectable when a 100-fold excess of unlabeled STAT1 and STAT3 oligonucleotide was present (data not shown). Treatment with GBE (100 μg/ml) or NAC significantly inhibited the activation of STAT1 and STAT3 induced by high glucose or by mannitol (Figures 5C and 5D). Treatment with the IL-6 neutralizing antibody also significantly reduced high-glucose- or mannitol-induced STAT1/3 activation (data not shown). Accordingly, IL-6 mediated, redox-related, STAT3 transcription pathways are critical to ICAM-1 induction in HAECs. GBE could attenuate high-glucose- or mannitol-induced ICAM-1 expression by inhibiting STAT1/3 activation.

## Discussion

This study reveals several major findings. First, treatment of HAECs with high glucose for 4 days significantly increased endothelial ICAM-1 expression, which in turn enhanced endothelial adhesiveness to monocytes. Second, long-term presence of high glucose stimulated endothelial IL-6 secretion via the redox-dependent mechanism, which may then induce STAT3 activation and consequent ICAM-1 expression. Third, in parallel to high glucose, mannitol exerted the similar effects on inducing endothelial adhesiveness. Fourth, pretreatment with GBE inhibited high-glucose- as well as mannitol-induced intracellular ROS production, IL-6 secretion, STAT3 activation, ICAM-1 accumulation, and consequent endothelial adhesiveness to monocytes. Fifth, in parallel to GBE, NAC also exerted the similar endothelial protection effects in the presence of high glucose or mannitol. Taken together, it is suggested that long-term presence of high glucose might induce STAT3-mediated ICAM-1-dependent endothelial adhesiveness to monocytes, which requires the osmotic-related redox-dependent IL-6 activation. Furthermore, GBE, an antioxidant herb medicine, could prevent high-glucose-induced endothelial inflammation mainly by inhibiting IL-6 activation.

Hyperglycemia is considered one of the major pathogenic factors for atherogenesis and the progression of atherosclerosis in diabetes mellitus [23]. However, the detailed molecular pathological mechanisms may be varied according to the different findings in previous *in vitro* and *in vivo* studies. In the current study, endothelial ICAM-1 expression was increased by chronic treatment with high glucose, resulting in the upregulation of endothelial adhesiveness to monocytes. Our findings agreed in part with the previous suggestion that ICAM-1 may play an essential role in mediating endothelial adhesiveness to monocytes [24]. Besides, in the present study, long-term presence of high glucose increased endothelial IL-6 expression at least in part via the oxidative related mechanisms, which may then contribute to the increase of endothelial adhesiveness to monocytes. These findings also agreed in part with that of previous studies with different study designs, in which IL-6 was shown to induce endothelial ICAM-1 expression [25] and oxidative stress could be involved in the induction of IL-6 and ICAM-1 in HAECs treated with intermittent high glucose [26]. Finally, in the current study, both high glucose and mannitol could induce endothelial IL-6 expression, which supports the previous suggestion that high glucose may increase endothelial ICAM-1 expression by a nonspecific osmotic effect [6]. More interestingly, such effects on endothelial ICAM-1 expression could be reversible and directly related to the presence of high glucose since it could disappear gradually after high-glucose medium was replaced by normal medium (please see Additional file 1: Figure S3). Most mammalian cells respond to changes in cellular volume with a net movement of water driven by a redistribution of salt and or small organic molecules [27,28]. However, it is still not known which receptor or sensor for osmotic effect may regulate cell volume as well as cell signal transduction in endothelial cell. One of the possible receptors is receptor tyrosine kinase receptors (RTKs). Growth control and apoptosis in mammalian cells are highly regulated by RTKs. A number of reports also implicated a role of RTKs in osmosensing and volume control as upstream regulators. That osmotic stress activates p38 MAPK and JNK were described in mammalian cells [29-31]. Accordingly, our study connected the complex findings of previous studies and helped to elucidate a whole picture of detailed mechanisms for high-glucose-induced endothelial adhesiveness that mimics early *in vivo* atherogenesis.

It was recently shown that Interleukin-6 (IL-6) plays a beneficial role in anti-inflammatory activity to against bacterial infections [32], and enhances insulin action immediately at early recovery [33,34]. However, it is well known that IL-6 is a multifunctional cytokine regulating cellular responses especially in inflammation. It has been suggested that diabetes-related oxidative stress may induce vascular IL-6 expression and activate IL-6R and gp130, which in turn activates the JAK2/STAT3 signaling cascade [35]. In the present study, STAT1 and STAT3 rather than AP-1 and NF-kB were activated by high glucose in HAECs. More importantly, piceatannol, a STAT1/3 inhibitor, but not fludarabine, a STAT1 inhibitor, significantly suppressed high-glucose-induced ICAM-1 expression in HAECs, suggesting the contribution of STAT3 pathways in ICAM-1 induction. It has been previously shown that IL-6 may activate endothelial STAT3 and ICAM-1 expression [25]. The present study further indicated that long-term presence of high glucose may induce endothelial IL-6 production by the osmotic-related redox-dependent mechanisms, which in turn activate endothelial ICAM-1 expression mainly via the STAT3 transcriptional pathways. Our findings support the rationale that strict glucose control may be critical to reduce osmotic injury and related oxidative stress for endothelial protection in clinical hyperglycemia.

There have been very few, mainly *in vitro,* evidence for the effects of GBE in the presence of high glucose [36]. To our knowledge, this is the first study demonstrating the direct endothelial protection effects of GBE in the presence of long-term high glucose. In this study, pretreatment of GBE could does-dependently suppressed HG-induced ICAM-1 accumulation in HAECs. ( Additional file 1: Figure S2) It was also shown that NAC, similar to GBE, could also reduce high-glucose-induced ROS generation, IL-6 secretion, and ICAM-1 accumulation in HAECs. Taken together, our findings suggest that GBE, as an antioxidant herb medicine, may inhibit IL-6 activation and consequent ICAM-1 expression probably by its antioxidant as well as anti-inflammatory effect. It was recently shown that GBE may have anti-oxidant ability by its free radical scavenger property [37] and mitochondrial uncoupling effect [38]. However, high dosage GBE (50–200 μg/mL) didn’t reduce hydrogen peroxide generation by mitochondria in rats heart [39], which may be due to the complex components of GBE and its selective uncoupling of mitochondrial oxidative phosphorylation. Future studies are required to validate the current findings in *in vivo* hyperglycemia.

The detailed mechanisms of the anti-inflammatory effects of GBE for vascular protection might be complex and varied in different experimental as well as clinical conditions. We have recently shown the contribution of Nrf-2-mediated heme oxygenase 1 induction to the inhibitory effects of GBE on TNF-α-induced VCAM-1 expression in endothelial cells [14]. In the present study, GBE was shown to inhibit high-glucose-induced ICAM-1 expression and consequent adhesiveness of HAECs. These findings are in line with our previous findings that GBE could inhibit cytokine-stimulated endothelial adhesiveness [13], vascular smooth muscle cell proliferation [40], and *in vivo* neointimal hyperplasia [12] mainly by its inhibitory effects on intracellular oxidative stress. Though the molecular mechanisms could be different, GBE may have similar anti-inflammatory effects in either TNF-α-induced or high-glucose-induced endothelial adhesiveness, suggesting the potential universal role of GBE in endothelial protection. However, given the complex and multiple effects of GBE shown in the current and previous studies [12-14], it is possible that other redox-independent mechanisms may be also involved in the beneficial effects of GBE on endothelial protection in the presence of high glucose. It might be important since the pathological changes are much more complex in *in vivo* hyperglycemia. Future *in vivo* studies are required to clarify this issue.

It should be indicated that GBE used in the current and our previous studies is the same preparation with fixed portions of standard components [12-14]. Though the individual effects of the major components of GBE, flavone glycoside and terpenlactones, on atherogenesis and vascular inflammation remain to be investigated, the dosage of GBE used in the current study is similar to or equivalent to that had been used in our previous *in vitro* and *in vivo* studies [12-14]. Furthermore, it was shown in the previous study that in the volunteers, after administration of GBE (160 mg) in free (Ginkgoselect) or phospholipid complex (Ginkgoselect Phytosome) forms, the maximum plasma concentrations, C(max), of total ginkgolides A, B and bilobalide were 85.0 and 181.8 ng/mL for Ginkgoselect and Ginkgoselect Phytosome, respectively. The C(max) values reached at 120 minutes for the free form and at 180–240 minutes for the phospholipid complex form [41]. In such case, the serum level seems similar to the concentration used in the current and previous studies. As that had been mentioned previously, the dose of GBE used in the current and previous studies may not be directly translated to that in clinical situations since there might be significant variety in individual drug metabolism and accumulation effects of such kind of herb medicine in different person [12,13]. However, the GBE and NAC used in the current study have also been widely and safely used clinically for years. Future *in vivo* studies or even clinical trials seem feasible to test the potential role and investigate the proper dose of GBE and other antioxidant/anti-inflammatory agents such as NAC for vascular protection especially in patients with chronic hyperglycemia and elevated serum IL-6 levels.

## Conclusions

Chronic exposure to high glucose can induce endothelial ROS production, stimulate IL-6 expression, activate STAT1 and STAT3, increase ICAM-1 expression, and enhance endothelial adhesiveness to monocytes at least in part by its osmotic effects. Treatment with GBE, a clinically used herbal antioxidant, may inhibit high-glucose-induced IL-6 release and ICAM-1 accumulation, leading to a reduction in endothelial adhesiveness to monocytes. Our findings might help to elucidate the complex molecular mechanisms of high-glucose-induced endothelial inflammation and provide the potential rationale to strict glucose control and potential antioxidant therapeutic strategy targeting on IL-6 for endothelial protection in the presence of hyperglycemia. Future *in vivo* study is still indicated for proper dosing before the above therapeutic strategy may be validated clinically.

## Competing interests

The authors declare that they have no competing interests.

## Authors’ contributions

Jia-Shiong Chen conducted the experiments and contributed to the study implementation, statistical analysis, interpretation, and the preparation of the manuscript. Yung-Hsiang Chen conducted the experiments and contributed to the study conception and design, implementation, and the preparation of the manuscript. Both JS Chen and YH Chen contributed equally to this paper. Po-Hsun Huang and Hsiao-Ya Tsai helped to conduct the experiments and contributed to the study conception and design, implementation, and interpretation. Yuh-Lien Chen contributed to the study conception and design, implementation, and interpretation. Shing-Jong Lin contributed to the study conception and design. Jaw-Wen Chen supervised the study conduction and contributed to the study conception and design, implementation, statistical interpretation, the preparation and finalization of the manuscript. All authors approved the final manuscript for publication.

## Supplementary Material

Additional file 1 Figure S1. Pretreatment with GBE had no significant effects on high-glucose-induced AP-1 and NF-κB activation in HEACs. Figure S2. Pretreatment with GBE does-dependently suppressed high glucose-induced ICAM-1 accumulation in HAECs. Figure S3. Endothelial ICAM-1 expression was increased by high-glucose (25 mM) stimulation for 4 days, which was losing after the replacement of normal glucose medium (5 mM) for 1–4 days. Click here for file
