# Supplementary material for: Ginkgo biloba extract reduces high-glucose-induced endothelial adhesion by inhibiting the redox-dependent interleukin-6 pathways
Source: Cardiovasc Diabetol. 2012 May 3;11:49. doi: 10.1186/1475-2840-11-49 (PMC3434011; doi:10.1186/1475-2840-11-49)
Supplement: Additional file 1 Figure S1. — Pretreatment with GBE had no significant effects on high-glucose-induced AP-1 and NF-κB activation in HEACs. Figure S2. Pretreatment with GBE does-dependently suppressed high glucose-induced ICAM-1 accumulation in HAECs. Figure S3. Endothelial ICAM-1 expression was increased by high-glucose (25 mM) stimulation for 4 days, which was losing after the replacement of normal glucose medium (5 mM) for 1–4 days. [file 1475-2840-11-49-S1.ppt]

## Slide 1
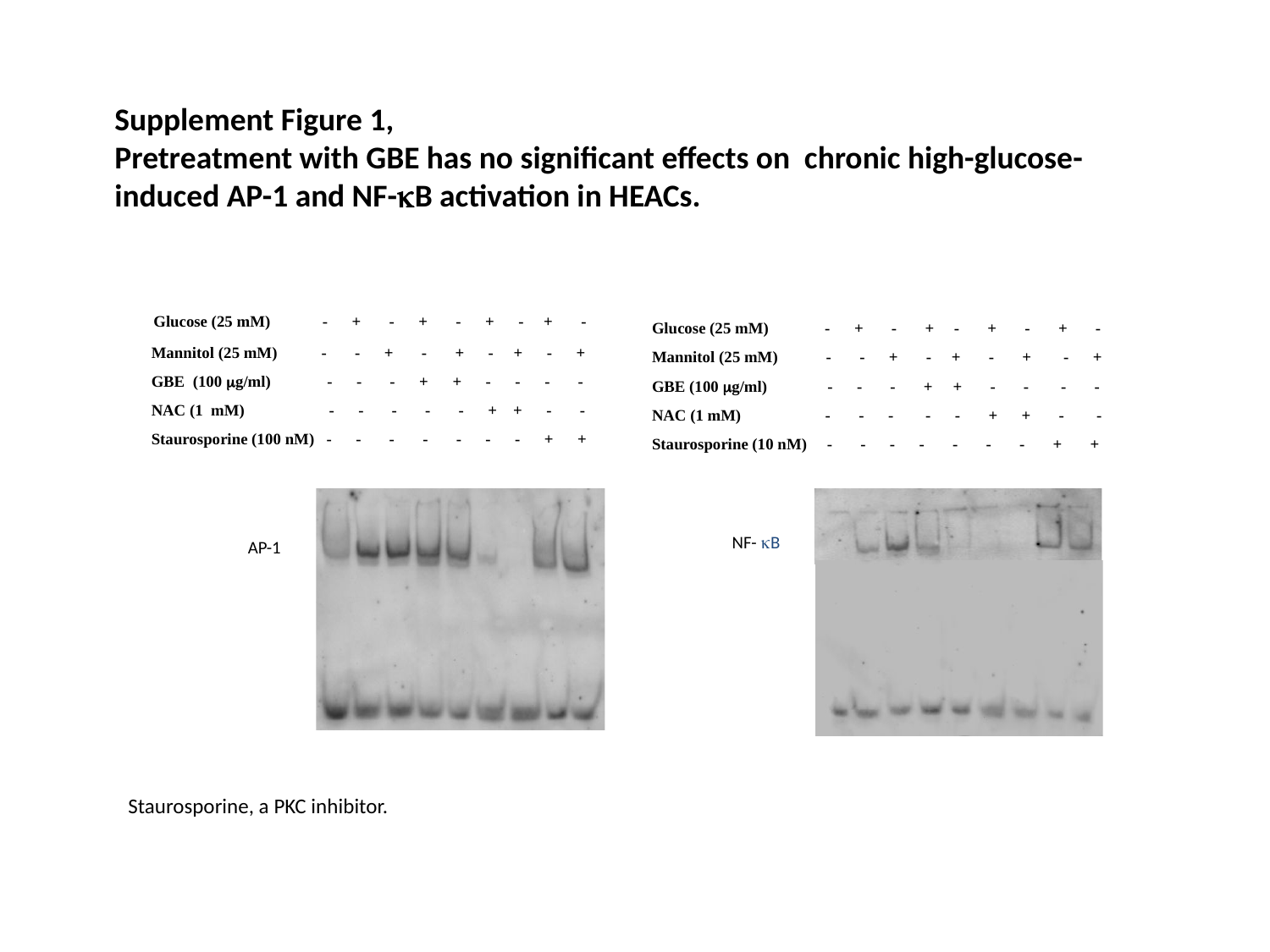

# Supplement Figure 1, Pretreatment with GBE has no significant effects on chronic high-glucose- induced AP-1 and NF-κB activation in HEACs.
 Glucose (25 mM) - + - + - + - + -
 Mannitol (25 mM) - - + - + - + - +
 GBE (100 μg/ml) - - - + + - - - -
 NAC (1 mM) - - - - - + + - -
 Staurosporine (100 nM) - - - - - - - + +
Glucose (25 mM) - + - + - + - + -
Mannitol (25 mM) - - + - + - + - +
GBE (100 μg/ml) - - - + + - - - -
NAC (1 mM) - - - - - + + - -
Staurosporine (10 nM) - - - - - - - + +
NF- B
AP-1
Staurosporine, a PKC inhibitor.

## Slide 2
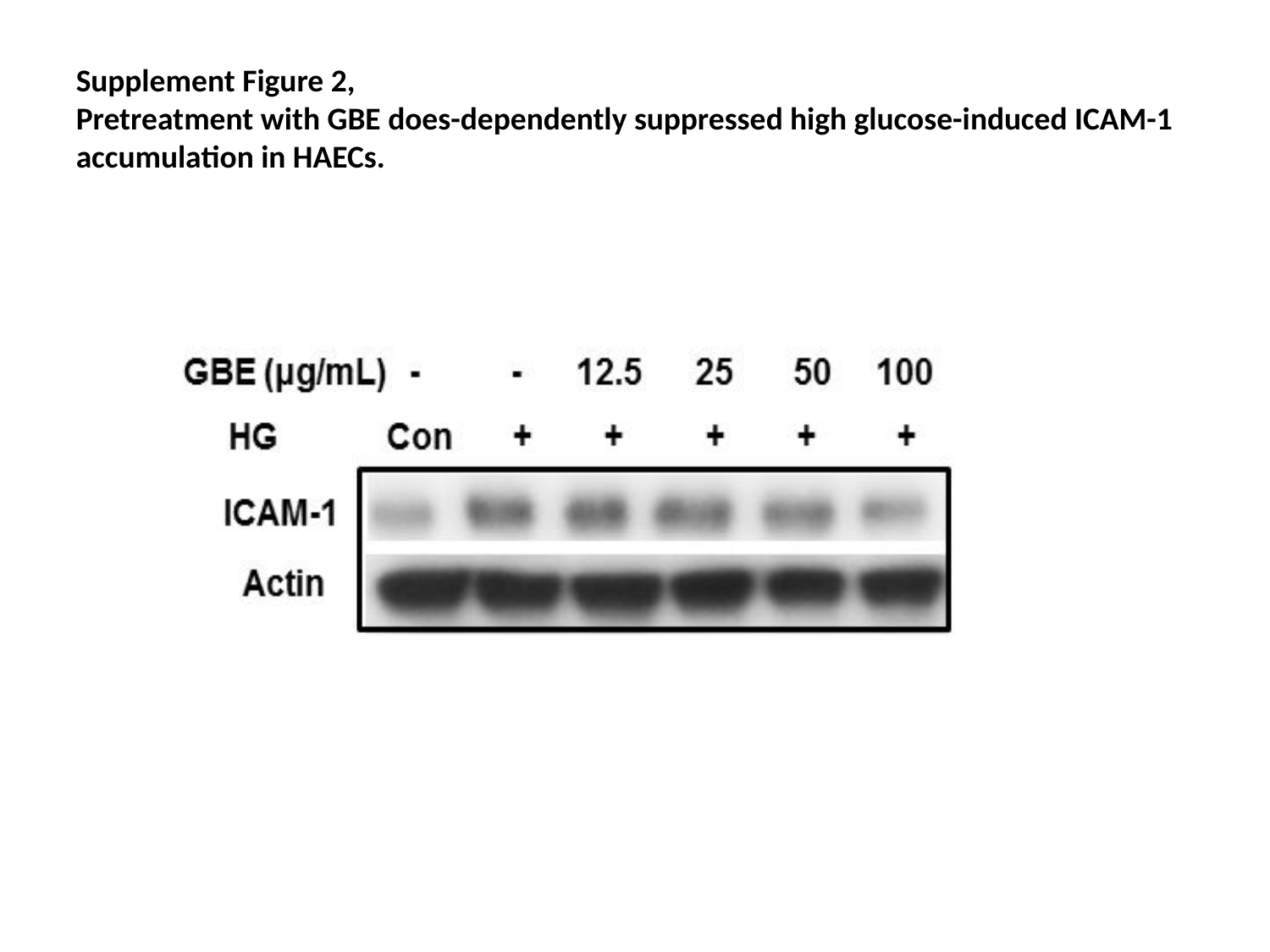

# Supplement Figure 2, Pretreatment with GBE does-dependently suppressed high glucose-induced ICAM-1 accumulation in HAECs.

## Slide 3
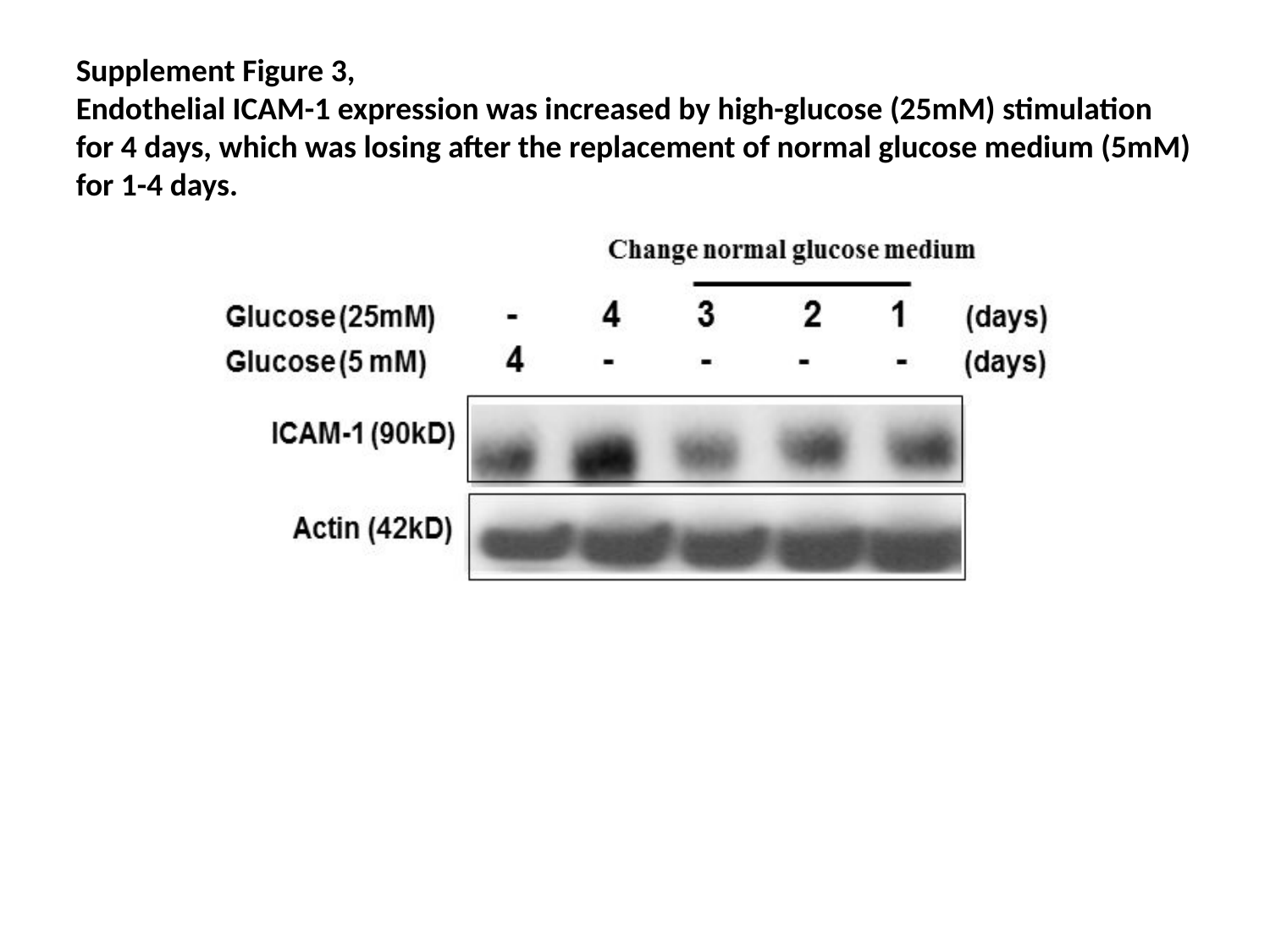

# Supplement Figure 3, Endothelial ICAM-1 expression was increased by high-glucose (25mM) stimulation for 4 days, which was losing after the replacement of normal glucose medium (5mM) for 1-4 days.
